# Dataset-Agnostic Biological Age Calculator and Predictions of Mortality in NHANES (1999-2010), UK Biobank (2006-2010), and Dalian Heart Failure (2015-2023) Cohorts: Development and Validation Study

**DOI:** 10.2196/95321

**Published:** 2026-10-20

**Authors:** Haipeng Liu, Angelina Amabi Perez Aranda, Anselm Au, Derek Wu, Haixin Zhou, Wing Tak Wong, Tong Liu, Mehrdad Shahmohammadi Beni, Gary Tse

**Affiliations:** 1 Cardiovascular Analytics Group, Kowloon, China (Hong Kong); 2 School of Nursing and Health Sciences, Hong Kong Metropolitan University, 1 Sheung Shing Street, Ho Man Tin, Kowloon, China (Hong Kong), +852 3970 2942; 3 Department of Biomedical Engineering and Health Sciences, McMaster University, Hamilton, ON, Canada; 4 School of Life Sciences, Chinese University of Hong Kong, Shatin, China (Hong Kong); 5 Tianjin Key Laboratory of Ionic-Molecular Function of Cardiovascular disease, Department of Cardiology, Tianjin Institute of Cardiology, Second Hospital of Tianjin Medical University, Tianjin, China

**Keywords:** biological age, numerical analysis, Klemera-Doubal method, KDM, principal component analysis, PCA, open-source model

## Abstract

**Background:**

A positive difference between biological age (BA) and chronological age indicates accelerated aging. The application of existing BA models is limited by high technical demands, a lack of alignment across diverse populations, and difficulty in updating them to accommodate expanding cohorts.

**Objective:**

To address the issues in existing tools, this project aims to develop and validate an automated toolkit for adaptive BA estimation.

**Methods:**

The proposed toolkit, named BACalc, facilitates the processing of raw clinical datasets stored in CSV format. Within the graphical user interface, users can select (1) the chronological age column, (2) binary variables (eg, sex, smoking, and alcohol use), and (3) continuous variables (eg, creatinine levels) for BA estimation, using the principal component analysis (PCA) method with the Dubina correction or the Klemera-Doubal method (KDM). All numerical operations are implemented using the NumPy and Pandas libraries, and linear modeling components are provided by scikit-learn and SciPy. The program generates three main output files: (1) the row-wise predictions, (2) the regression statistics, and (3) the explicit linear equations and PCA loadings. Example outputs were generated using the National Health and Nutrition Examination Survey (NHANES, 1999‐2010), UK Biobank (2006‐2010), and Dalian Heart Failure (2015‐2023). The results were compared with the reference BioAge R package.

**Results:**

Using the KDM model, the BACalc-derived and BioAge-derived estimates exhibit a near-perfect correlation (*r*=0.9937, mean absolute error 2.02 y). The PCA-Dubina model also shows a strong correlation with BioAge (*r*=0.8871), within a ±20-year window of accelerated aging residuals. In the UK Biobank, a 1-year increase in PCA-Dubina–derived age acceleration was associated with a 1.77% higher hazard of all-cause mortality (hazard ratio [HR] 1.018, 95% CI 1.017‐1.018; *P*<.001). Similarly, KDM-derived age acceleration was associated with a 1.59% higher hazard of mortality per year of accelerated aging (HR 1.016, 95% CI 1.016‐1.016; *P*<.001). In the Dalian cohort, a 1-year increase in age acceleration derived from the PCA-Dubina model (BAc) independently predicted mortality with a 2.84% increased hazard per year (HR 1.028, 95% CI 1.022‐1.035; *P*<.001). Similarly, KDM-derived age acceleration (BA_EC) was associated with a 1.26% increased hazard of mortality per year of accelerated aging (HR 1.013, 95% CI 1.007‐1.018; *P*<.001).

**Conclusions:**

BACalc provides a reliable platform for BA estimation, consistent with previously validated computational frameworks and convenient for real-time updating. BA acceleration predicted mortality in 2 independent cohorts.

## Introduction

As the global population continues to age, it is essential to develop optimized approaches to manage chronic diseases, strengthen health care infrastructure, and promote healthy aging [1], where comprehensive, accurate, and patient-specific estimation of aging status plays a key role. Biological age (BA) refers to the composite physiological state of the organism and/or its various systems [2]. A positive difference between BA and chronological age (CA) indicates accelerated aging. Its estimation is important as BA and (BA – CA) can be used for predicting longevity [3], adverse outcomes [4], and for prognostication [5].

Different models have been proposed to estimate BA based on biomarkers of different mechanisms, including DNA methylation, telomere length changes, proteomics, metabolomics, and the combination of heterogeneous biomarkers [6]. Regarding the algorithm, multiple linear regression and principal component analysis (PCA) methods have been used in processing multiple factors, while the Klemera and Doubal’s method (KDM) provides a more accurate estimation by adjusting the weights of different variables according to their correlations with age [7]. With the recent development of AI, machine learning and deep learning models including deep neural networks, convolutional neural networks, and deep convolutional long-term memory have been introduced to process multidimensional data in BA studies [7,8].

Despite these advances, there are three major limitations that preclude the widespread use of BA models: (1) the high technical demands needed to develop the models [9], (2) lack of alignment of BA models across diverse populations [10], and (3) difficulties in updating the models when new data are received or cohorts are expanded [11]. Thus, the aim of this project is to develop a tool that can address the issues above by simplifying and automating BA model development and updates.

## Methods

### Program Structure

The present toolkit, named BACalc, facilitates the ingestion of raw clinical datasets stored in CSV format. Multiple CSV files can be loaded and merged row-wise to form a unified dataset. Within the graphical interface, users can select (1) the CA column, (2) optional binary variables (eg, sex, smoking, drinking, etc), and (3) continuous variables (for biomarkers) used for BA estimation. When a binary split variable is defined, the full dataset is partitioned into 2 groups, and the full pipeline is run independently within each group, provided that at least a minimum number of participants are available. In this study, we developed BACalc, a BA calculator that can process raw clinical datasets stored in CSV format. Within the graphical interface, users can select (1) the CA column, (2) binary variables (eg, sex, smoking, and alcohol use), and (3) continuous variables (eg, creatinine levels), used for BA estimation. All numerical operations are implemented via the NumPy and pandas libraries, and linear modeling components are provided by scikit-learn and SciPy. This vectorized implementation allows for rapid processing of typical clinical datasets, while the graphical user interface layers provide an accessible interface for users without the need of scripting or coding. The present toolkit implements 2 methods, PCA method with Dubina correction and the KDM. Both methods operate on the same preprocessed biomarker matrix and CA vector. The program generates a total of three main output files for every user-defined group: (1) the row-wise predictions, (2) the regression statistics, and (3) the explicit linear equations and PCA loadings. Example outputs were generated using the publicly available National Health and Nutrition Examination Survey (NHANES).

The program includes an optional feature to compute mean arterial pressure (MAP) when columns containing systolic blood pressure (SBP) and diastolic blood pressure (DBP) are present. In those cases, MAP is computed according to the standard approximation:


(1) $$MAP=\frac{1}{3}SBP+\frac{2}{3}DBP$$

It can be included as an additional biomarker in the subsequent modeling steps.

### Calculation of BA

The present toolkit implements 2 methods for BA calculation: the PCA method with Dubina correction and the KDM. Both methods operate on the same preprocessed biomarker matrix and CA vector.

### Data Preprocessing and Standardization

#### Overview

Prior to model fitting, all selected variables are converted to numeric form. Any nonnumeric entries are set to missing values. For each biomarker *j* within a given analysis group, missing entries are imputed using the median of that biomarker in that group. This simple and robust imputation strategy avoids the introduction of bias toward extreme values while retaining all available participants for modeling.

Let *x_ij_* denote the observed value of biomarker *j* for participant *i* in a group with *N* participants and *m* biomarkers. For each biomarker, the mean *μ_j_* and SD *σ_j_* are computed as follows:

. (2) $$\mu_{j}=\frac{1}{N}\sum_{i=1}^{N}x_{ij},\;\sigma_{j}=\sqrt{\frac{1}{N}\sum_{i=1}^{N}{\left(x_{ij}-\mu_{j}\right)}^{2}}$$

To avoid division by 0, biomarkers with zero variance are assigned *σ*_*j*_=1. The standardized (*z*-scored) biomarker matrix *z_ij_* is then defined as follows:


(3) $$z_{ij}=\frac{x_{ij}-\mu_{j}}{\sigma_{j}}$$

This standardization step ensures that all biomarkers contribute on a comparable scale, regardless of their original units or ranges.

#### Method 1: PCA-Dubina Algorithm

The initial part of the computation involves the generation of a biological age score (BAS). As raw biomarker data varied significantly in units and magnitude, the program first performed standardization. PCA is then performed on the standardized biomarker matrix *Z*, and the first principal component (PC1) is extracted.

Let *l* = (*l*_1_,...,*l_m_*) denote the loading vector corresponding to PC1. For each participant *i*, the PC1 score is:


(4) $$PC1_{i}=\sum_{j=1}^{m}l_{j}z_{ij}$$

To ensure that higher scores correspond to higher CA, the present implementation automatically corrects the directionality of PC1 by inspecting its Pearson correlation with CA. If corr(PC1,CA) <0, the loading vector *I* is multiplied by −1 so that PC1 is positively associated with age.

The BAS for participant *i* was then calculated directly in the original biomarker space as a linear combination of the input biomarkers *x_ij_* weighted by the component loadings normalized by the biomarker SD. Specifically, we defined weights


(5) $$w_{j}=\frac{l_{j}}{\sigma_{j}}$$

and an intercept term *w*_0_ chosen so that the projection is centered:


(6) $$w_{0}=-\sum_{j=1}^{m}w_{j}\mu_{j}$$

The governing equation for BAS in the present model is therefore:


(7) $${BAS}_{i}=w_{0}+\sum_{j=1}^{m}w_{j}x_{ij}$$

By construction, BAS is dimensionless. Let *μ*_BAS_ and *σ*_BAS_ denote the mean and SD of BAS in the training population, and let *μ*_CA_ and *σ*_CA_ denote the mean and SD of CA in the same group. To convert the dimensionless BAS into a timescale (y), a T-scale transformation is applied:


(8) $${BA}_{i}=\left({BAS}_{i}-\mu_{BAS}\right)\cdot \left(\frac{\sigma_{CA}}{\sigma_{BAS}}\right)+\mu_{CA}$$

This transformation maps the distribution of BA to match the central tendency and spread of CA in the training sample.

However, BA estimates derived from regression or PCA often exhibit a regression to the mean effect, where older individuals appear younger, whereas younger individuals appear older. To rectify this, the Dubina correction was applied [12]. The slope *b* of the regression of the initial BA against CA is calculated as follows:


(9) $$b=\frac{\sum_{i=1}^{N}\left({BA}_{i}-\mu_{BA}\right)\left({CA}_{i}-\mu_{CA}\right)}{\sum_{i=1}^{N}{\left({CA}_{i}-\mu_{CA}\right)}^{2}}$$

where *μ*_BA_ is the mean of the uncorrected BA values in the training group. The corrected biological age, denoted BA_c_, was then given by:


(10) $${BA}_{c,i}={BA}_{i}+\left({CA}_{i}-\mu_{CA}\right)\cdot \left(1-b\right)$$

This step forced the slope of the predicted age against CA to unity, ensuring that deviations from the diagonal represented genuine biological acceleration or deceleration instead of artifacts of regression shrinkage. For interpretability, the program also reported the BA acceleration derived from the PCA-Dubina pipeline:


(11) $${Accel}_{{BA}_{c,i}}={BA}_{c,i}-{CA}_{i}$$

This quantifies how many years older (positive values) or younger (negative values) a participant appears biologically relative to their CA.

#### Method 2: KDM

The second module uses the KDM, which assumes that BA is a latent variable driving the linear deterioration of biomarkers. For each biomarker *j*, a simple linear model is fitted with CA as the predictor:


(12) $$x_{ij}=k_{j}\cdot {CA}_{i}+q_{j}+\epsilon_{ij}$$

where *k_j_* is the slope, *q_j_* is the intercept, and *∈_ij_* are residuals. These parameters are estimated via ordinary least squares regression. The root mean square error (RMSE) for biomarker *j* is given by


(13) $$s_{j}=\sqrt{\frac{1}{N}\sum_{i=1}^{N}{\left(x_{ij}-{\hat{x}}_{ij}\right)}^{2}}$$

where ${\hat{x}}_{ij}=k_{j}{CA}_{i}+q_{j}$ is the model prediction. In the implementation, *s_j_* is constrained to a small positive minimum to avoid division by 0. For each biomarker, the Pearson correlation coefficient *r_j_* between *x_j_* and CA is determined.

The algorithm first computes the BA estimate (BA*_E_*) for each participant, which aggregates the contributions of all biomarkers. A weight *w_j_* is assigned to each biomarker, defined as the square of the signal-to-noise ratio:


(14) $$w_{j}={\left(\frac{k_{j}}{s_{j}}\right)}^{2}$$

For participant *i*, the KDM BA estimate BA_E_*_,i_* was then computed as the weighted average of the ages predicted by each individual biomarker:


(15) $${BA}_{E,i}=\frac{\sum_{j=1}^{m}w_{j}\left(\frac{x_{ij}-q_{j}}{k_{j}}\right)}{\sum_{j=1}^{m}w_{j}}$$

In addition, our program implements a variance-based adjustment to calculate the corrected biological age (BA_EC_). This requires the estimation of the characteristic correlation (*r*_char_) of the biomarker set. Based on the individual correlations *r_j_*, the characteristic correlation is computed by:


(16) $$r_{\text{char}}=\frac{\sum_{j=1}^{m}\frac{r_{j}^{2}}{\sqrt{1-r_{j}^{2}}}}{\sum_{j=1}^{m}\frac{r_{j}}{\sqrt{1-r_{j}^{2}}}}$$

with numerical safeguards applied when |*r_j_*| is extremely small or close to unity.

This metric was then used to estimate the population variance of BA, $S_{BA}^{2}$. In the implementation, 2 components are considered. First, the variance of the difference between the KDM estimate and CA is computed:


(17) $${\mathrm{Var}}_{\Delta }=\mathrm{Var}\left({BA}_{E}-CA\right)$$

Second, the variance of CA is approximated from its range:


(18) $$\hat{Var}\left(CA\right)\approx \frac{{\left(\max_{i}{CA}_{i}-\min_{i}{CA}_{i}\right)}^{2}}{12}$$

Following Klemera and Doubal, a correction term that depends on *r*_char_ and the number of biomarkers *m* is then subtracted:


(19) $$S_{BA}^{2}=\max\left(S_{\min}^{2},{\mathrm{Var}}_{\Delta }-\frac{1-r_{char}^{2}}{r_{char}^{2}}\cdot \frac{\hat{\mathrm{Var}}\left(CA\right)}{m}\right)$$

where $S_{min}^{2}$ is a small positive floor (set to 0.1 in the present implementation) to avoid unrealistically small or negative variance estimates.

Finally, for each participant *i*, the corrected KDM BA BA_EC_*_,i_* is computed by combining the biomarker-based BA estimate (BA_E_*_,i_*) with CA (CA*_i_*) in a Bayesian fashion:


(20) $${BA}_{EC,i}=\frac{{BA}_{E,i}\cdot \left(\sum_{j=1}^{m}w_{j}\right)+\left(\frac{{CA}_{i}}{S_{BA}^{2}}\right)}{\left(\sum_{j=1}^{m}w_{j}\right)+\left(\frac{1}{S_{BA}^{2}}\right)}$$

This formulation effectively reduces the prediction error by anchoring the estimate to the CA when the biomarker data are noisy or incoherent, while still allowing large deviations when the biomarker pattern strongly supports them.

Analogous to the PCA-Dubina pipeline, the KDM module reports a BA acceleration measure based on the corrected KDM estimate:


(21) $$\text{Acce}{\text{l}}_{{BA}_{\text{EC,i}}}={BA}_{EC,i}-{CA}_{i}$$

### Program Output and Computational Scheme

The program generates three main output files for each user-defined group: (1) the row-wise predictions, (2) the regression statistics, and (3) the explicit linear equations and PCA loadings. The predictions file stores, for each participant, the CA, the intermediate BAS, the PCA-derived BA and BA_c_, the KDM-derived BA_E_ and BA_EC_, and the corresponding acceleration measured (BA*_c_ –* CA and BA_EC_ – CA). The statistics file contained global goodness-of-fit metrics for each method (PCA-Dubina and KDM), including the coefficient of determination (*R*^2^), Pearson correlation coefficient (*r*), RMSE, and mean absolute error (MAE) between each BA estimate and CA.

For a given method, let *y_i_* denote the CA of participant *i* and ${\hat{y}}_{i}$ the corresponding estimated BA (eg, BA_c_*_,i_* or BA_EC,_*_i_*). The coefficient of determination was calculated using:


(22) $$R^{2}=1-\frac{\sum_{i=1}^{N}{\left(y_{i}-{\hat{y}}_{i}\right)}^{2}}{\sum_{i=1}^{N}{\left(y_{i}-\bar{y}\right)}^{2}}$$

where $\bar{y}$ is the mean of *y_i_*. The RMSE and MAE are given by:


(23) $$RMSE=\sqrt{\frac{1}{N}\sum_{i=1}^{N}{\left(y_{i}-{\hat{y}}_{i}\right)}^{2}}$$

(24) $$MAE=\frac{1}{N}\sum_{i=1}^{N}\left|y_{i}-{\hat{y}}_{i}\right|$$

Pearson correlation coefficient between *y_i_* and ${\hat{y}}_{i}$ is reported:


(25) $$r=\frac{\sum_{i=1}^{N}\left(y_{i}-\bar{y}\right)\left({\hat{y}}_{i}-\bar{\hat{y}}\right)}{\sqrt{\sum_{i=1}^{N}{\left(y_{i}-\bar{y}\right)}^{2}\sqrt{\sum_{i=1}^{N}{\left({\hat{y}}_{i}-\bar{\hat{y}}\right)}^{2}}}}$$

with $\bar{\hat{y}}$ being the mean of the estimated ages.

The generation of explicit equations allows users to apply the trained models externally without rerunning the optimization pipeline, which is useful in the development of portable apps on mobile devices where computational resources are rather limited. For each group, the program writes a human-readable summary that includes the PCA-based BAS and BA equations, the Dubina-corrected BA_c_ equation as a function of CA and biomarker values, and the full set of KDM parameters (*k_j_*, *q_j_*, *s_j_*, and *w_j_*) together with the final BA_E_ and BA_EC_ formulas. In addition to tabular outputs, the program generated plots for graphical illustration. For each group, scatterplots of BA_c_ vs CA and BA_EC_ vs CA are generated, together with identity lines and fitted regression lines with 95% confidence bands.

Beyond predictive accuracy, the computational efficiency of BACalc was a primary design priority. By leveraging the Python NumPy library for high-performance vectorized operations and implementing compact linear models, BACalc achieves significant speed advantages. Even when processing extensive clinical datasets (n>10,000), the system is capable of training and evaluation in a matter of seconds on standard hardware, making it highly scalable for large-scale epidemiological studies. The BACalc source code and executable can be downloaded freely from [13].

### Model Validation and Performance Evaluation

To validate the robustness and accuracy of the proposed model, we used the NHANES dataset as curated by the BioAge R package, with no additional inclusion or exclusion criteria [14]. This validation process incorporated a comprehensive panel of 13 physiological biomarkers: albumin, alkaline phosphatase, blood urea nitrogen, creatinine, hemoglobin A_1c_ (HbA_1c_), C-reactive protein (CRP), lymphocytes, mean corpuscular volume, glucose, red cell distribution width, total cholesterol, white blood cell (WBC) count, and SBP. Using this standardized feature set, we benchmarked the BA estimates generated by our implementation of the KDM and the PCA-Dubina method against the established KDM projections from the BioAge R package. Furthermore, we have ensured median imputation in the BioAge R package so that it matches the default median imputer implemented in BACalc. To empirically evaluate the impact of imputation choice, we performed a sensitivity analysis on the NHANES dataset using 4 commonly used imputation techniques: median, mean, k-nearest neighbors (k=5), and iterative imputation (MICE [multiple imputation by chained equations]) [15]. The NHANES dataset comprised 55,081 participants, taken from the BioAge R package repository.

To further validate our tool, we conducted a comprehensive Bland-Altman agreement and residual analysis against the established BioAge R package, applying both tools to the same unweighted cohort to ensure a direct, one-to-one methodological comparison. We compared the BACalc KDM implementation (BA_EC) against the standard KDM in the R package, and similarly compared the BACalc PCA-Dubina model (BAc) against the standard. To analyze the sensitivity of the proposed method to age and sex, we then evaluated the calibration across distinct age bins (<40, 40‐60, and >60 y) with sub-group analysis between genders.

### Additional Datasets for Predictive Validity Evaluation

To validate the clinical relevance of BACalc estimates, we assessed the association between BA acceleration (delta age=BA-CA) and longitudinal clinical outcomes in 2 independent datasets.

We used a retrospective population-based clinical cohort comprising 2781 patients hospitalized with heart failure from the First Affiliated Hospital of Dalian Medical University (2015‐2023). To comprehensively capture multisystem aging, BACalc estimates were generated using a 20-biomarker clinical panel. This included markers of cardiovascular/hemodynamic stress (SBP, DBP, B-type natriuretic, and high-sensitivity cardiac troponin I), renal function (creatinine, urea, and uric acid), metabolic/lipid profiles (glucose, total cholesterol, triglycerides, low-density lipoprotein-C, and high-density lipoprotein-C), liver function (alanine aminotransferase, aspartate aminotransferase, and total bilirubin), and hematological/immune status (WBC, neutrophils, hemoglobin, red blood cells [RBC], and platelets). We used Cox proportional hazards models, controlling for CA at baseline, sex, smoking history, and drinking history, with administrative right-censoring applied to surviving patients. We calculated BA acceleration and linked these estimates to longitudinal clinical outcomes using fully adjusted Cox proportional hazards models and Kaplan-Meier survival analyses.

In addition, we evaluated the predictive validity on the large-scale UK Biobank (UKBB) dataset. We included 501,936 participants with available biomarker data at the initial assessment center visit (assessment instance 0) and linked mortality follow-up. The biomarkers include renal function (creatinine, urea, urate, and cystatin C), metabolic/lipid profiles (glucose, total cholesterol, triglycerides, low-density lipoprotein [direct], high-density lipoprotein-C, and HbA_1c_), liver function (alanine aminotransferase, aspartate aminotransferase, alkaline phosphatase, CRP, albumin, total bilirubin, and total protein), and hematological/immune status (WBC, hemoglobin, RBC, nucleated RBC, platelets, monocytes, neutrophils, lymphocytes, eosinophil, hematocrit percentage, mean corpuscular volume, and mean corpuscular hemoglobin concentration). BA acceleration was calculated using 29 blood count and biochemistry biomarkers measured at this visit, and 54,507 all-cause deaths were observed during follow-ups.

### Ethical Considerations

This study used existing deidentified and anonymized data in the public domain directly downloadable from the NHANES website. Thus, according to the Common Rule, this study was exempt from the institutional review board review and the informed consent requirement. NHANES was conducted by the Centers for Disease Control and Prevention National Center for Health Statistics. NHANES survey procedures and protocols were approved by the National Center for Health Statistics ethics review board for each survey cycle [16].

The UKBB analysis was performed under application number 79146 as part of a wider study for predictive modeling for cardiovascular disease. The UKBB was approved by the North West Multi-Centre Research Ethics Committee (number 11/NW/0382) and conducted in accordance with the Declaration of Helsinki.

For the dataset from the First Affiliated Hospital of Dalian Medical University, this study was a retrospective analysis of existing clinical data and did not involve any interventions or interactions with participants. The study was conducted in accordance with the Declaration of Helsinki and was approved by the Ethics Committee of the First Affiliated Hospital of Dalian Medical University (approval number PJ-KS-KY-2024‐616). As this was a retrospective study using anonymized data, informed consent was waived by the Ethics Committee.

## Results

### Initial Validation of the BACalc Model

Benchmarking studies demonstrate that the BACalc model generates BA estimations in close alignment with the BioAge R package (Figure 1). This high level of congruency validates the algorithmic integrity of the BACalc implementation. Specifically, when evaluating the KDM model, the BACalc estimations exhibit a near-perfect correlation with the BioAge R package results (*r*=0.9937), accompanied by an MAE of only 2.02 years. Furthermore, the PCA-Dubina model within the BACalc framework shows strong statistical agreement with the R package reference, yielding a correlation coefficient of 0.8871. When analyzing the variance across these methodologies, the data distribution of the BACalc KDM model more closely mirrors that of the BioAge R package than the PCA-based approach. Notably, the accelerated age residuals representing the divergence between BA and CA remained largely within a ±20-year window for both the BioAge R package and the BACalc PCA-Dubina model, with the highest data density concentrated within this range.

**Figure 1.**
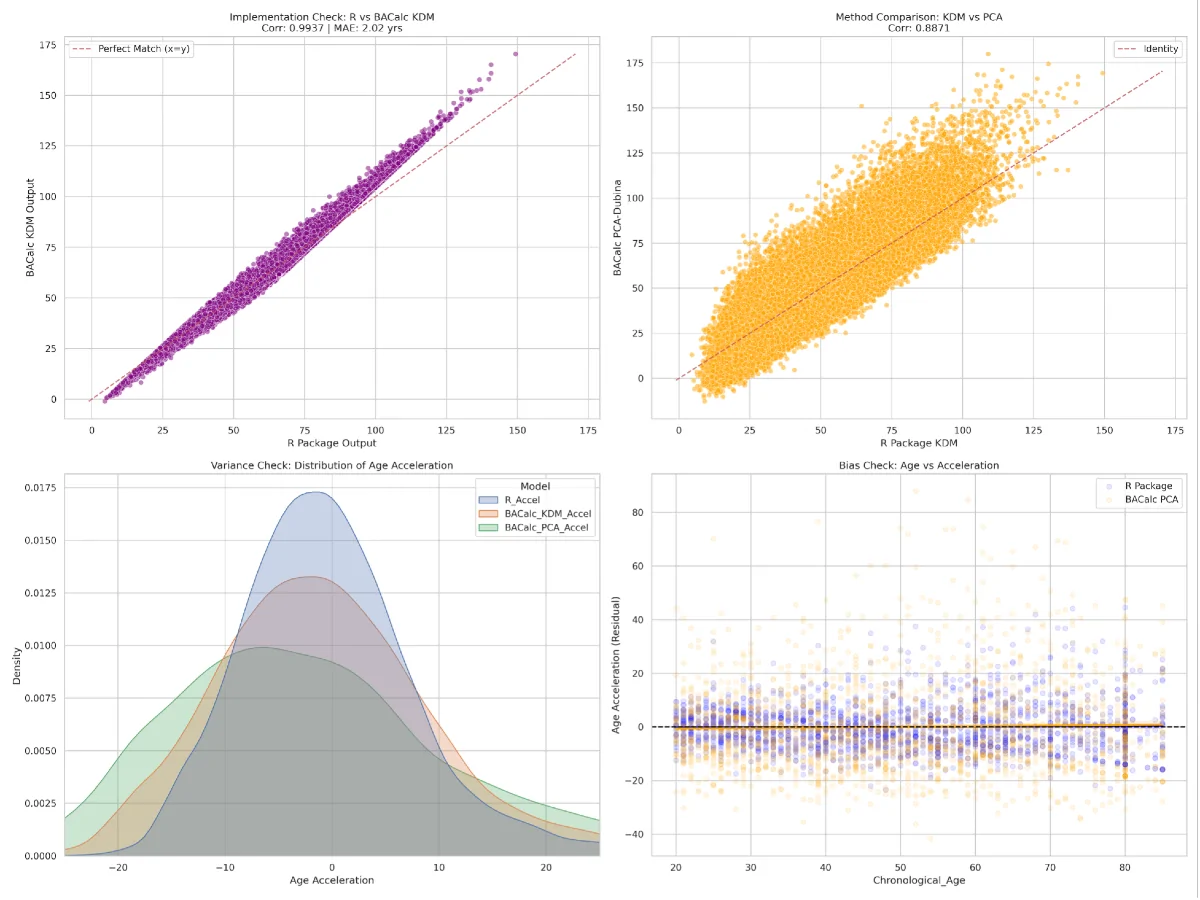
Correlation analysis comparing the Klemera-Doubal method (KDM) biological age predictions generated by BACalc and the BioAge R package [14] using the National Health and Nutrition Examination Survey (NHANES) IV dataset. MAE: mean absolute error; PCA: principal component analysis.

### Sensitivity Analysis of Imputation Methods

This dataset reflects a realistic epidemiological scenario with an overall missingness of 9.79% (ranging from ~9% for blood pressure to 29.69% for CRP). As shown in Figure 2, BA predictions remained highly robust regardless of the imputation method used. The predictions across all 4 methods exhibited near-perfect correlation (Pearson *r*>0.991 for all pairwise comparisons). The density distributions of predicted ages (BAc and BA_EC) and the variance of age acceleration were visually and statistically indistinguishable across the median, mean, k-nearest neighbors, and MICE techniques.

**Figure 2.**
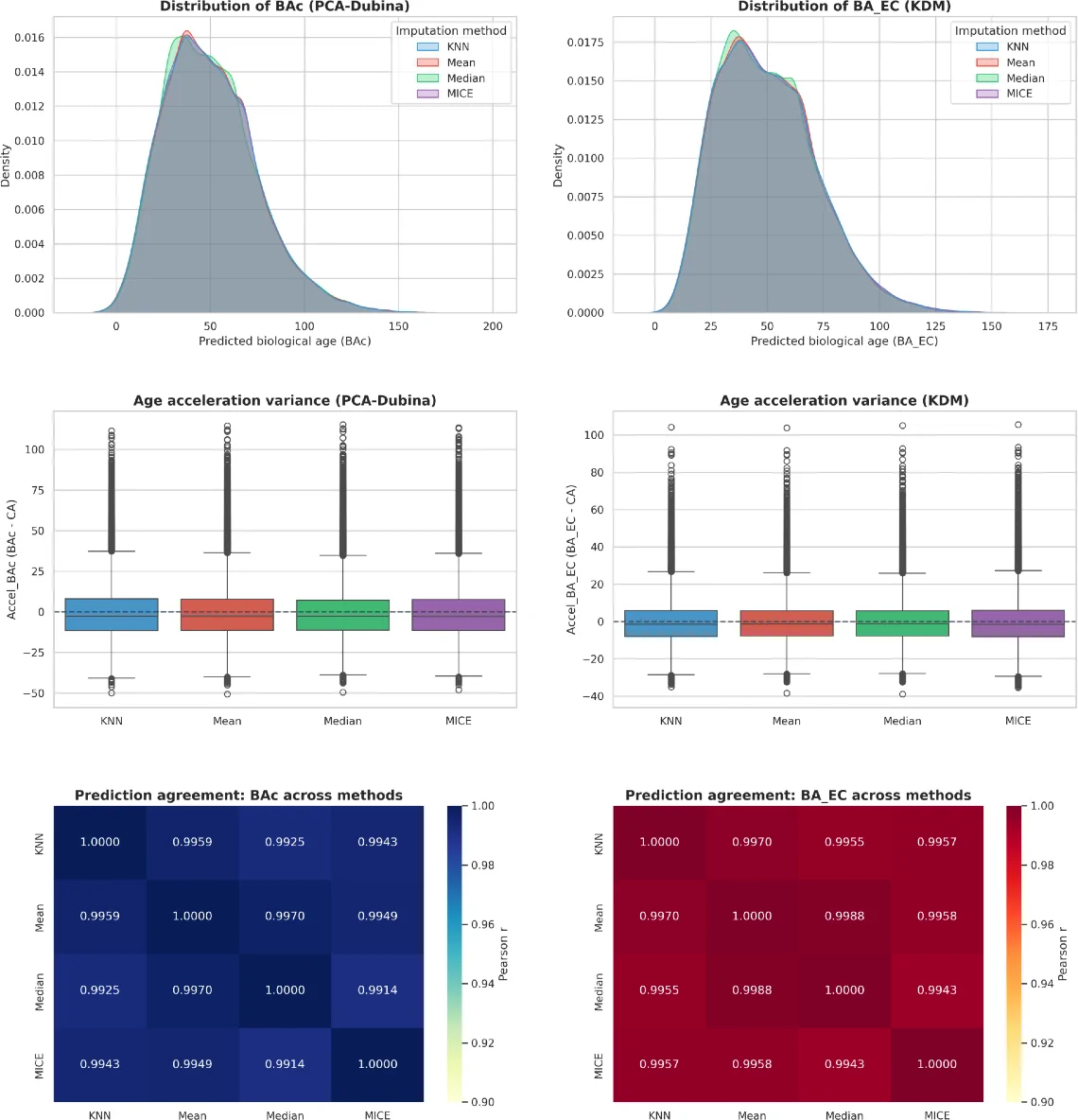
Sensitivity analysis on the effect of missing data imputation method (k-nearest neighbors [KNNs], mean, median, and MICE [multiple imputation by chained equations]) on the results. KDM: Klemera-Doubal method; PCA: principal component analysis.

### KDM vs PCA-Dubina: Comparison With CA

The comparative performance of the 2 methods, PCA-Dubina and KDM, implemented in BACalc is shown in Figure 3. When evaluated against CA, the KDM approach demonstrated superior predictive precision, achieving a higher coefficient of determination (*R^2^*=0.597) and correlation coefficient (*r*=0.844) compared to the PCA method (*R^2^*=0.174; *r*=0.740).

**Figure 3.**
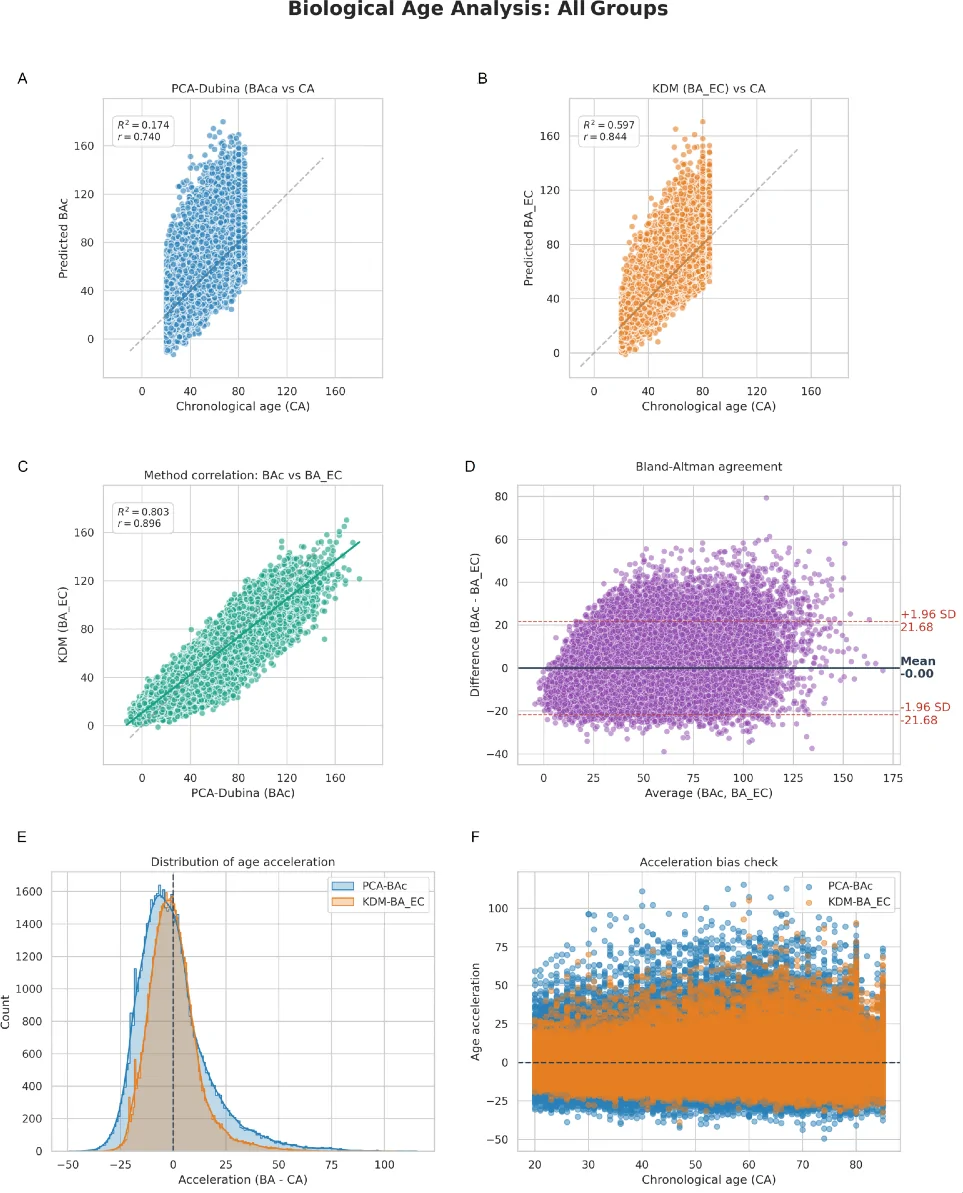
Comparative analysis of biological age (BA) estimation methods in BACalc. (A-B) The linear regression results of BAs derived from BACalc Klemera-Doubal method (KDM) and principal component analysis (PCA) against chronological age (CA); (C) the regression between the BAs derived from BACalc KDM and PCA; (D) and (E) the distribution of aging acceleration and its dispersion against CA.

There is a strong, positive correlation between the 2 BA estimates (*R^2^*=0.803; *r*=0.896). The Bland-Altman agreement analysis further elucidates this relationship, showing a mean difference of 0.00 years, indicating no systematically fixed bias between the 2 estimators. However, the limits of agreement are notably wide (±21.68 y), implying substantial random variation in individual predictions. Furthermore, the discrepancy between the 2 methods tends to increase as the average BA increases.

### KDM vs PCA-Dubina: Comparison With Standard KDM

The Bland-Altman analysis demonstrated exceptional calibration between the BACal and standard KDM models, yielding an overall mean bias of 0.000 years and highly constrained limits of agreement ([−10.68, 10.68] y; Figure 4A). The overall MAE was 4.14 years, with an RMSE of 5.45 years, confirming high precision between the 2 models. As shown in Figure 4C, the residuals are normally distributed and centered around 0.

Between BACalc PCA-Dubina model (BAc) and the standard KDM, the limits of agreement widen to [−21.51, 21.51] years (Figure 4B), with an RMSE of 10.97 years. This illustrates the fundamental mathematical divergence between a variance-based paradigm (PCA) and a regression-based paradigm (KDM) when assessing physiological decline. Crucially, despite this expected individual-level variance, the 2 algorithms maintain an overall mean bias of 0.000 years.

Regarding the calibration across distinct age ranges (<40, 40‐60, and >60 y). For both methods (KDM and PCA in BACalc), the mean bias against standard KDM in BA estimation remained near-absolute zero across all age groups (eg, maximum age-group bias for PCA vs KDM was merely 0.42 y). Subgroup analysis by gender revealed only a marginal mean difference (males: −2.05 y; females: +1.90 y). These robust agreement metrics confirm that BACalc replicates biological aging methodologies with high mathematical fidelity and remains highly stable across all demographic subgroups without systematic drift.

**Figure 4.**
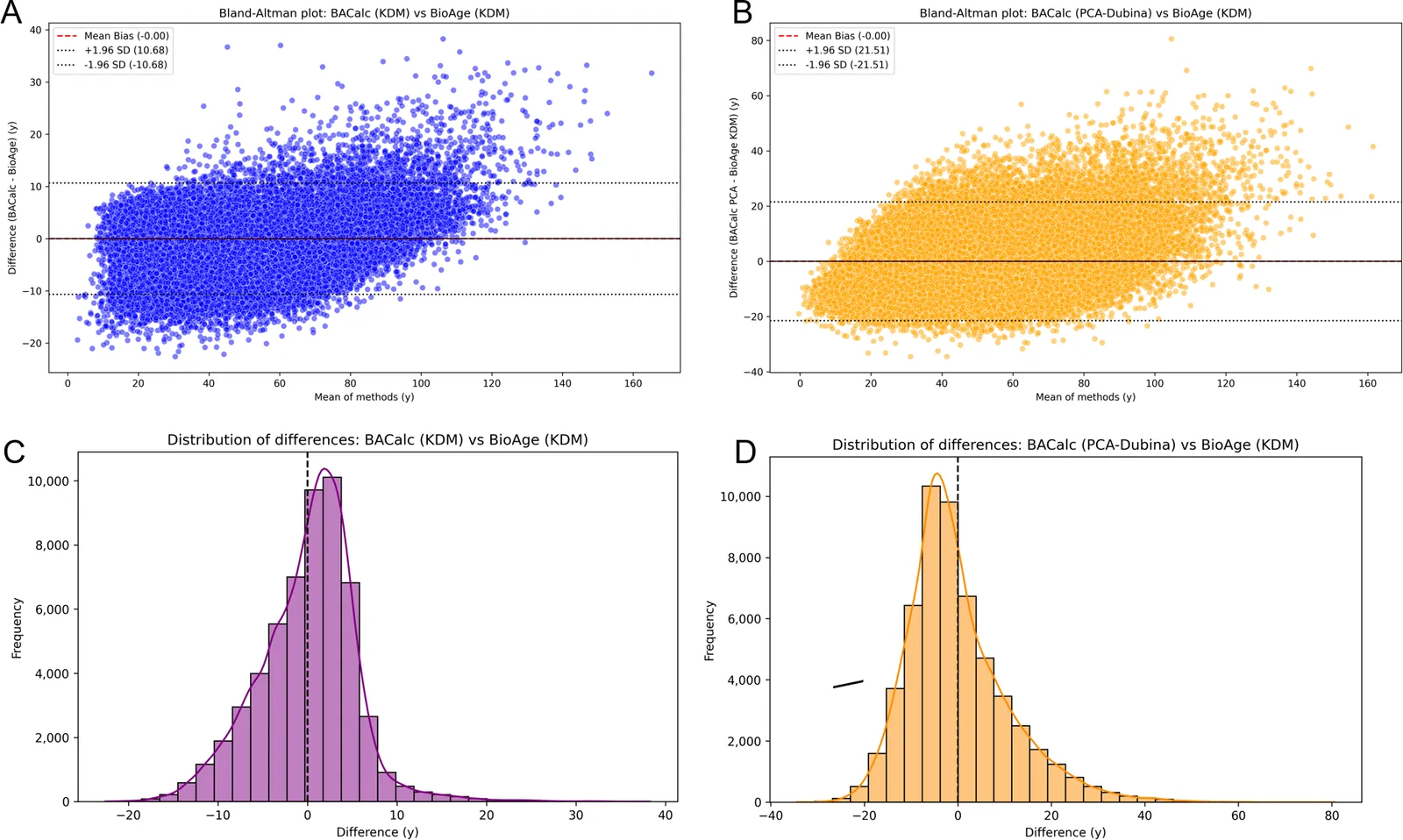
Comparative analysis of biological age (BA) estimation errors of Klemera-Doubal method (KDM) and principal component analysis (PCA) methods in BACalc. (A) and (B) the Bland-Altman results; (C) and (D) the distribution of estimation errors.

### Explicit Linear Equations

The explicit linear equations derived from the BACalc model provide a quantitative framework for assessing biological aging through the integration of 13 key biomarkers. Equation (26) illustrates the multivariate regression model. Notably, biomarkers associated with metabolic dysregulation (HbA_1c_ and glucose) and inflammation (CRP) exhibit positive coefficients, contributing to an acceleration of the BA estimate. Conversely, markers of synthetic liver function, such as albumin, display negative coefficients, consistent with the age-associated decline in hepatic protein synthesis.

Table 1 further decomposes these relationships using the KDM parametrization. The specific weights assigned to each biomarker reflect their relative reliability in predicting CA within the NHANES cohort. SBP and blood urea nitrogen emerged as highly weighted parameters, suggesting that vascular and renal stiffness are most associated with aging in this specific model.

**Table 1.** Klemera-Doubal method (KDM) parameters for explicit equations generated by the BACalc model.

Biomarker	*k* (slope)	*q* (intercept)	Weight
Albumin	−0.002527	4.348	5.521×10^−5^
Alkaline phosphatase	0.1383	64.15	4.087×10^−5^
Blood urea nitrogen	0.1131	7.822	0.0005984
Creatinine	0.003724	0.6939	0.0002474
Hemoglobin A_1c_	0.01395	4.946	0.0003087
Log-normalized C-reactive protein	0.0006252	0.2306	6.166×10^−6^
Lymphocytes	−0.04888	32.63	3.644×10^−5^
Mean corpuscular volume	0.05397	86.79	0.0001006
Glucose	0.3578	80.71	0.0001787
Red cell distribution width	0.01022	12.62	8.125×10^−5^
Total cholesterol	0.183	186.3	2.18×10^−5^
Systolic blood pressure	0.4536	102	0.0007404
White blood cell count	−0.009997	7.715	2.469×10^−5^

In contrast, although inflammatory markers, such as CRP, show a steep slope (ie, *k*), they are assigned lower weights, likely due to higher interindividual variance. Finally, equation 28 applies a corrective transformation to the calculated BA (ie, BA_E_). This step integrates a CA anchor to mitigate the characteristic regression to the mean bias often observed in biological clocks, thereby improving prediction accuracy at the extremes of the age range.


(26) $$\begin{array}{rl} {BA}_{c}= & -60.999\\ +(0.58257\times CA)\\ +(-9.8184\times albumin)\\ +(0.15875\times alkaline\;phosphatase)\\ +(0.61715\times blood\;urea\;nitrogen)\\ +(9.804\times creatinine)\\ +(6.6414\times hemoglobin\;A_{1c})\\ +(14.637\times log\;normalized\;C\;reactive\;protein)\\ +(-0.27053\times lymphocytes)\\ +(-0.34334\times mean\;corpuscular\;volume)\\ +(0.18261\times glucose)\\ +(3.3212\times red\;cell\;distribution\;width)\\ +(0.0032436\times total\;cholesterol)\\ +(0.17593\times systolic\;blood\;pressure)\\ +(1.1794\times white\;blood\;cell\;count) \end{array}$$

(27) $$BA_{E}=\frac{\sum \left({Weight}_{i}\times \frac{{Biomarker}_{i}-q_{i}}{k_{i}}\right)}{\sum \left({Weight}_{i}\right)}$$

(28) $${BA}_{EC}=\frac{(BA_{E}\times 0.0024406)+\left(\frac{CA}{397.1}\right)}{0.0024406+\left(\frac{1}{397.1}\right)}$$

The BACalc model generated explicit linear equations that successfully capture the multisystem nature of biological aging. The model parameterization (Table 1) reveals that renal function and vascular health are the most heavily weighted predictors of age in the NHANES dataset. The resulting algorithm integrates these weighted physiological inputs with a CA correction to produce a BA estimate that is both physiologically interpretable and mathematically stable despite variance in individual biomarkers.

### Predictive Validity of BACalc Estimates for Clinical Outcomes

Both models generated by BACalc demonstrated highly significant predictive validity for all-cause mortality using 2 additional datasets. Thus, on the UKBB dataset, both biological age acceleration measures generated by BACalc were significantly associated with all-cause mortality in unadjusted Cox models in the UKBB. A 1-year increase in PCA-Dubina–derived age acceleration was associated with a 1.77% higher hazard of all-cause mortality (hazard ratio [HR] 1.018, 95% CI 1.017‐1.018; *P*<.001). Similarly, KDM-derived age acceleration was associated with a 1.59% higher hazard of mortality per year of accelerated aging (HR 1.016, 95% CI 1.016‐1.016; *P*<.001; Figure 5).

**Figure 5.**
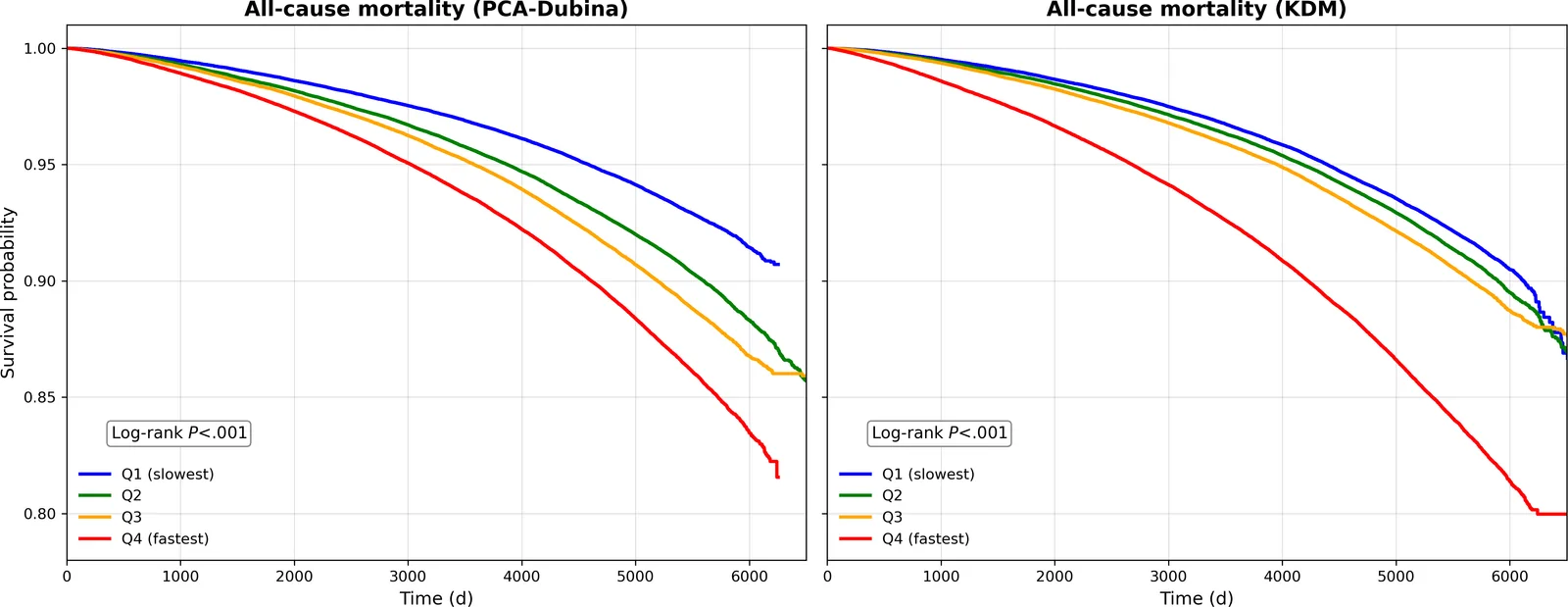
Application of BACalc-derived aging acceleration using PCA-Dubina and KDM in predicting all-cause mortality on the UK Biobank (UKBB) dataset of normal population. KDM: Klemera-Doubal method; PCA: principal component analysis.

In the Dalian heart failure cohort, a 1-year increase in age acceleration derived from the PCA-Dubina model (BAc) independently predicted mortality with a 2.84% increased hazard per year (HR 1.028, 95% CI 1.022-1.035; *P*<.001). Similarly, KDM-derived age acceleration (BA_EC) was associated with a 1.26% increased hazard of mortality per year of accelerated aging (HR 1.013, 95% CI 1.007-1.018; *P*<.001). Kaplan-Meier survival analysis demonstrated clear risk stratification for mortality based on BACalc estimations. Across both the PCA-Dubina and KDM models, participants in the highest quartile of age acceleration (Q4, fastest aging) exhibited significantly lower overall survival probabilities compared to those in the lowest quartile (Q1, slowest aging; Figure 6). These distinct survival trajectories for all-cause mortality were highly statistically significant (log-rank *P*<.001), visually underscoring the prognostic capability of the toolkit.

**Figure 6.**
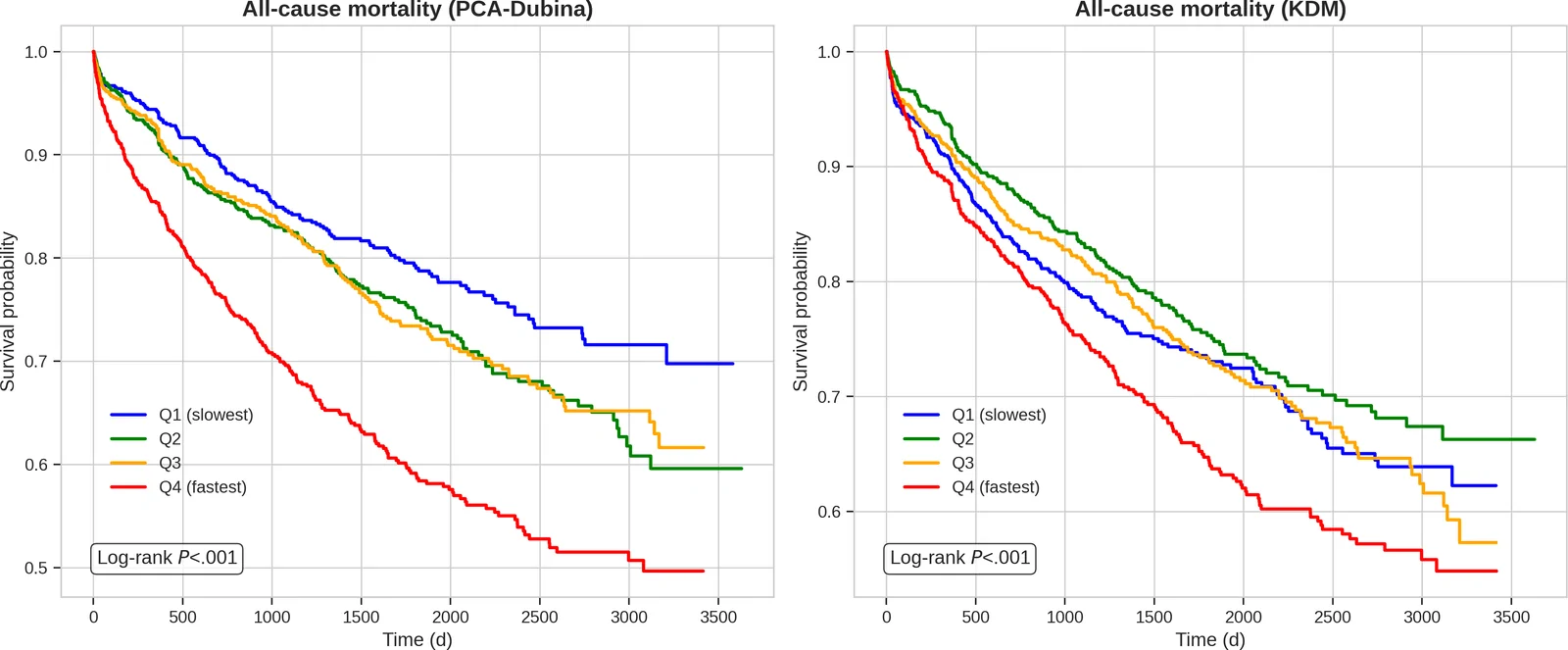
Application of BACalc-derived aging acceleration using PCA-Dubina and KDM in predicting all-cause mortality on the Dalian dataset of patients hospitalized for heart failure. KDM: Klemera-Doubal method; PCA: principal component analysis.

On the UKBB dataset, both BA acceleration measures generated by BACalc were significantly associated with all-cause mortality in unadjusted Cox models. A 1-year increase in PCA-Dubina–derived age acceleration was associated with a 1.77% higher hazard of all-cause mortality (HR 1.018, 95% CI 1.017‐1.018; *P*<.001). Similarly, KDM-derived age acceleration was associated with a 1.59% higher hazard of mortality per year of accelerated aging (HR 1.016, 95% CI 1.016‐1.016; *P*<.001; Figure 6).

## Discussion

### KDM vs PCA-Dubina

The BACalc model generates BA estimations in close alignment with the BioAge R package. When evaluating the KDM model, the BACalc estimates exhibit a near-perfect correlation with the BioAge R package results (*r*=0.9937), accompanied by an MAE of only 2.02 years. The PCA-Dubina model within the BACalc framework also shows strong statistical agreement with the R package reference, yielding a correlation coefficient of 0.8871. The accelerated age residuals, representing the divergence between BA and CA, remained largely within a ±20-year window for both the BioAge R package and the BACalc PCA-Dubina model, with the highest data density concentrated within this range.

While both KDM and PCA-Dubina methods show a positive linear relationship with CA, the PCA-Dubina model exhibits significantly higher variance and dispersion around the regression line, particularly in older age groups (Figure 3A-D), suggesting less stability in its predictions relative to the KDM model.

The distribution of age acceleration highlights distinct characteristics in how these models quantify aging. The PCA method produces a platykurtic distribution with a wider spread of acceleration values compared to the more leptokurtic distribution of the KDM (Figure 3E). Importantly, the acceleration bias check reveals systematic bias in the PCA method; as CA increases, the PCA-derived age acceleration shows a positive upward trend. In contrast, the KDM model displays a consistent, flat distribution across the age spectrum, indicating that it has reduced residual bias and likely serves as a more robust metric for biological aging independent of time.

PCA models are simple and straightforward and can therefore be used for screening aging markers or solving covariance problems, whereas the assumption of linearity limits the accuracy [17]. In comparison, the KDM models improve accuracy by adjusting the weights of variances. Although KDM models have a higher computational burden, they are more accurate and stable with less dispersion and distortion at either end of the age spectrum [18]. Based on our results and the conclusions of existing studies, we recommend that KDM models are preferable to PCA ones especially when BA has a wide spectrum and heterogeneous biomarkers are included.

Regarding the predictive validity, the aging accelerations derived from both KDM models showed high performance on the datasets of patients with cardiovascular diseases and normal population. The BA-related biomarkers in these datasets cover the liver, renal, and immune functions and comprehensively reflect the health status of the participants [19]. The survival curves on the Dalian dataset of patients who are hospitalized differ from the UKBB ones in shape. The former indicates that the mortality rate was high since the observation, while the latter reflects the common trend that deaths happen at higher ages in the general population. Therefore, both KDM models show the potential to predict long-term health status in both patients and healthy participants. An earlier study on a smaller UKBB subset showed that the biomarkers in KDM-based BA models significantly improved the prediction of mortality and hospital admissions over CA alone [20]. A recent study on 2 large Chinese cohorts showed that KDM-based BA could predict mortality independently from CA and other potential confounders [5]. Therefore, by lowering the technical barrier for BA estimation, our proposed tool may provide a potential tool of not only BA estimation but also clinical risk prediction, particularly for health researchers and public health practitioners who may not be familiar with R-based tools.

### Analysis of Aging Factors: Neglected Long-Term Health Issues

Some well-established biomarkers such as blood urea nitrogen and creatinine are reflected in our results [21], while their relationships with aging might be nonlinear [22]. Meanwhile, the results revealed some aging-related biomarkers that are often neglected, including HbA_1c_, mean corpuscular volume, and glucose. HbA_1c_ and glucose levels reflect the risk of metabolic disorder and diabetes, which is a lasting and increasing public health problem especially in lower-income countries [23], where the pathological changes during prediabetes and multiple complications consist of a major challenge in the aging population. Mean corpuscular volume has recently been proposed as a predictive marker for colorectal or esophageal cancer, chronic renal disease, and ischemic stroke or coronary procedures [24]. Further investigation on these factors in different cohorts can indicate the aging acceleration for different organs and systems.

### Limitations and Future Directions

Despite the good performance of BACalc, there are several methodological constraints: (1) the current implementation is limited to cross-sectional datasets and relies on linear imputation using the median for missing values, which may introduce bias when missingness is systematic or when biomarkers exhibit skewed distributions; (2) both the PCA-Dubina and KDM pipelines assume linear relationships between individual biomarkers and CA, which can obscure biologically relevant nonlinear or interaction effects; and (3) the present model currently supports only a single modal set of blood-based chemistry variables, restricting its applicability to cohorts that have incorporated emerging omics layers (eg, DNA methylation [25], proteomics [26], and metabolomics [27], or imaging-derived phenotypes [28]).

Future development will address these gaps by extending BACalc to longitudinal analyses, enabling the estimation of individual aging trajectories and the computation of rate of change metrics that are less susceptible to regression-to-the-mean artifacts. Incorporating flexible nonlinear modeling frameworks such as generalized additive models [29], penalized splines [30], machine learning, or neural networks [31] will allow the capture of curvature and interaction effects while preserving interpretability. We also plan to integrate a modular multiomics interface, wherein users can supply heterogeneous biomarker matrices and BACalc will automatically harmonize scales, perform feature selection, and combine modalities through stacked ensemble learning [32]. Finally, to improve robustness to missingness, advanced imputation strategies (MICE and matrix completion) and uncertainty propagation will be implemented.

### Conclusions

We developed an open-source and versatile dataset-agnostic model named BACalc; this model employs PCA-Dubina and KDM models for predicting BA from biomarkers and CA. We have validated our model using previous methods and obtained good agreement. BACalc has the ability to accurately estimate BA from biomarkers, produce detailed plots, and provide explicit linear equations for a specific user-supplied cohort. The present toolkit and its open-source nature will be useful in reducing discrepancies in BA estimation through a transparent and reproducible framework.

## Abbreviations

BA: biological age
BAS: biological age score
CA: chronological age
CRP: C-reactive protein
DBP: diastolic blood pressure
HbA_1c_: hemoglobin A_1c_
HR: hazard ratio
KDM: Klemera-Doubal method
MAE: mean absolute error
MAP: mean arterial pressure
MICE: multiple imputation by chained equations
NHANES: National Health and Nutrition Examination Survey
PCA: principal component analysis
RBC: red blood cells
RMSE: root mean square error
SBP: systolic blood pressure
UKBB: UK Biobank
WBC: white blood cell
